# Real‐World Assessment of Liver Corrected T1 and Magnetic Resonance Elastography in Predicting Liver Disease Progression

**DOI:** 10.1111/liv.70280

**Published:** 2025-08-14

**Authors:** Kathleen E. Corey, Nabih Nakrour, Emily D. Bethea, Jessica E. Shay, Karin L. Andersson, Irun Bhan, Lawrence S. Friedman, Avinash R. Kambadakone, Laura E. Dichtel, Raymond T. Chung, Mukesh Harisinghani

**Affiliations:** ^1^ Gastroenterology Division, Liver Center Massachusetts General Hospital Boston Massachusetts USA; ^2^ Department of Medicine Harvard Medical School Boston Massachusetts USA; ^3^ Radiology Department Massachusetts General Hospital Boston Massachusetts USA; ^4^ Department of Medicine Newton‐Wellesley Hospital Newton Massachusetts USA; ^5^ Department of Medicine Tufts University School of Medicine Boston Massachusetts USA; ^6^ Neuroendocrinology Department Massachusetts General Hospital Boston Massachusetts USA

**Keywords:** chronic liver disease, noninvasive tests, patient management, prognostic, real‐world

## Abstract

**Background:**

Clinical guidelines emphasise identifying patients at risk of chronic liver disease progression. To avoid biopsy drawbacks, noninvasive imaging tests (NITs) have become part of standard‐of‐care. We assessed the real‐world clinical profile, referral trends, and use of magnetic resonance imaging (MRI)‐based tests, multiparametric MRI (mpMRI) and magnetic resonance elastography (MRE), as part of chronic liver disease management.

**Methods:**

Patients referred for abdominal imaging as part of standard‐of‐care were eligible for inclusion irrespective of liver aetiology or referral pathway. Liver fibrosis was assessed using MRE and disease severity using mpMRI (disease activity [iron‐corrected T1, cT1], liver fat content [LFC] and iron). *T*‐tests were used for group comparisons; Kaplan–Meier analyses for disease progression and area under the receiver operating characteristic (AUC) for diagnostic accuracy.

**Results:**

Over 18 months, 256 patients (53 years, 51% female, 48% with BMI > 30 kg/m^2^) were referred for liver imaging. The majority (66%) had steatotic liver disease (SLD). Of those with low MRE (73%) and low FIB‐4 (42%), 36% had elevated cT1 (> 875 ms). Those with MRE > 5 kPa had cT1 > 875 ms. During follow‐up, those with low MRE (< 3.14 kPa) but elevated cT1 (> 800 ms) had significant disease worsening (HR: 3.1, *p* = 0.0035) compared to all others. In the SLD group, cT1 (AUC: 0.71) outperformed LFC (AUC: 0.64) and MRE (AUC: 0.53) in predicting disease progression.

**Conclusion:**

Regardless of aetiology, patients with low fibrosis risk (MRE) but high disease activity (cT1) face a three‐times higher risk of progression. Integrating both biomarkers into standard care, especially for SLD, can guide management adjustments.

**Summary**

- Patients with low fibrosis (MRE) but elevated disease activity (corrected T1) are at risk (HR: 3.1) of disease progression over 12 months.
- Blood marker panels remain the primary tool for longitudinal management, while liver biopsy is rarely used in real‐world practice.
- Gastroenterology and Hepatology specialists are the leading referrers of MRI‐based NITs as part of routine clinical care.

## 1 Introduction

Current clinical practice guidelines highlight the importance of identifying patients at risk for advanced liver fibrosis due to the heightened risk for adverse outcomes, including cirrhosis and liver‐related mortality [1]. Chronic liver diseases, of which metabolic dysfunction–associated steatotic liver disease (MASLD) has one of the most rapidly increasing incidences and prevalences, are linked to severe and costly clinical outcomes such as cardiovascular disease and liver‐related mortality [2]. However, in many places, the reimbursement, integration, and implementation of regulatory‐cleared, effective, cost‐efficient, and noninvasive diagnostic tools for early detection and patient stratification remain limited [3]. This limitation contributes to a significant proportion of undiagnosed patients, which complicates timely intervention and management strategies and worsens the epidemic of advanced liver disease. For instance, despite the significant rise in investments funding the development and validation of imaging‐based noninvasive liver disease assessment (NILDA) techniques in the United States and Europe, the annual financial burden for MASLD, largely comprising liver and cardiovascular‐related morbidity and mortality, exceeds $103 billion and €35 billion annually, respectively [4].

Although histologic assessment via liver biopsy remains essential for identifying and evaluating the stage of diseases with specific hallmarks (e.g., autoimmune hepatitis), it has largely been supplanted for addressing disease severity of more homogenous diseases by noninvasive tests. This change is due primarily to the fact that percutaneous liver biopsy is a procedure being invasive, costly, and prone to both interpretation and sampling variability [5]. Recent clinical guidelines have recognised this and have provided diagnostic criteria for a diagnosis of MASLD without the requirement for a liver biopsy [6]. Several noninvasive tests (NITs), broadly separated into blood‐based and imaging‐based biomarkers (NILDAs), are currently used in clinical practice; however, some still face challenges in terms of specificity, sensitivity, and reproducibility. For instance, both the Fibrosis‐4 (FIB‐4) index and vibration‐controlled transient elastography (VCTE), which are typically recommended as a two‐step first‐line investigation strategy in MASLD clinical pathways, have notable limitations regarding patient stratification in some vulnerable populations. FIB‐4, a widely used marker for predicting significant fibrosis, performs inconsistently in certain populations, such as those with diabetes mellitus and younger individuals, and often fails to capture earlier disease stages like steatosis and inflammation [7]. In addition, due to its association with age, different FIB‐4 cut‐offs need to be used for different populations [8, 9] VCTE has also shown similar inconsistencies and variability [10, 11, 12], especially in young individuals with type‐2 diabetes mellitus [10, 11], as well as high variability. Therefore, there is a critical need for more accurate noninvasive alternatives to better stratify patients at risk of disease progression.

In specialist care, MRI‐based tools such as magnetic resonance elastography (MRE) and multiparametric magnetic resonance imaging (mpMRI) are being used to support patient management. MRE has shown good performance for identifying and staging liver fibrosis and cirrhosis, but poorer performance in identifying at‐risk metabolic dysfunction‐associated steatohepatitis (MASH) (also known as fibrotic MASH) [13, 14].

Liver cT1 is a proprietary Liver cT1 mpMRI biomarker that correlates with histological fibrosis and inflammation, predicts adverse liver and cardiac outcomes, and displays low inter‐observer variability, high repeatability [15, 16] and low coefficient of variation for disease monitoring [16]. These technical and clinical properties offer it several advantages over other commonly used NITs. Liver cT1 is delivered/provided via the FDA‐cleared LiverMultiScan software (Perspectum, Oxford, UK). In addition to cT1 showing good performance in identifying at‐risk disease and predicting adverse outcomes in population‐based studies [2, 17, 18], in real‐world management, cT1 has been used to prescribe resmetirom [19] and identify at‐risk patients requiring earlier intervention [20].

Both tests have been shown to be cost effective in the MASLD/MASH pathway (MRE: incremental cost‐effectiveness ratio (ICER) of $7048/QALY [21] and mpMRI: ICER of $5198/QALY [22]). However, there have been no real‐world head‐to‐head studies between these two techniques to assess how they perform in terms of identifying patients at risk of disease progression.

In this study, we investigated the use of NITs, primarily MRE and mpMRI, in clinical practice in a large academic medical centre. The aim was to understand the clinical profile, referral trends, and real‐world application of NITs in a real‐world setting to support management of patients with chronic liver diseases referred for imaging.

## 2 Methods

### 2.1 Study Design and Procedures

This was a real‐world retrospective longitudinal study of adult patients referred for abdominal imaging as part of standard‐of‐care (SoC) at Massachusetts General Hospital (MGH) from February 2022 to July 2024. Patients, regardless of liver disease aetiology, underwent routine quantitative imaging in addition to clinical assessment. All data was extracted by manual chart review and included patient demographics, comorbid conditions, baseline and follow‐up laboratory values, diagnostic imaging metrics, and pathology results. All clinical investigations were conducted following the principles of Good Clinical Practice (GCP) and in accordance with the Declaration of Helsinki and Istanbul, and informed consent was obtained from all participants. Approval was granted by the Mass General Brigham review board.

### 2.2 Image Acquisition, MRI Protocol and Reimbursement

All participants underwent non‐contrast abdominal MRI using the LiverMultiScan protocol (Perspectum, Oxford, UK) to assess disease activity (corrected T1 [cT1], liver fat content [LFC measured using proton density fat fraction; MRI‐PDFF] and liver iron concentration [LIC]). As part of the same acquisition protocol, 2D magnetic resonance elastography (MRE; Resoundant, USA) was also acquired for assessment of liver stiffness. All MR scans were performed on either a 3T GE Signa Premier or Siemens 3T VIDA Fit scanner with participants lying supine on a scanner and following a standard 4‐h fast. MRI scanner‐specific acquisition protocols for LiverMultiScan and elastography were used. The average complete scan time was < 25 min.

For LiverMultiScan (mpMRI) image acquisition, four transverse slices obtained at the porta hepatis location in the liver were acquired for each participant using a shortened modified look‐locker inversion (shMOLLI) and a multi‐echo spoiled gradient‐echo sequence to quantify T1, iron (LIC) and fat (LFC). cT1, LFC and LIC maps were delineated into whole liver segmentation maps using a semi‐automatic method that detects and removes non‐parenchymal structures and image artefacts. Pixel values from these segmentation maps were averaged, and median values were reported in the subsequent report provided. All LiverMultiScan analyses were performed during post‐processing by trained data analysts who were blinded to all clinical data.

For MRE, the Quantitative Imaging Biomarkers Alliance (QIBA) criteria were followed, and a region of interest (ROI) in the liver was drawn in the four central slices on the magnitude images to determine the liver stiffness. Voxels where the viscoelastic properties could not reliably be determined were excluded from the ROI. The ROI was drawn on the elastogram images using the magnitude images as an anatomic reference, avoiding the liver borders, fissures, gallbladder fossa and large vessels. All MRE analyses were performed in the Radiology Department at MGH using vendor‐supplied MRE processing software to automatically generate elastograms (wave images and stiffness maps).

All MRI scans were read for incidental findings following local site standards of practice. All procedures were reimbursed as per American Medical Association (AMA) healthcare payer policies using Current Procedural Terminology (CPT) codes 0648T and 0649T for LiverMultiScan, and 76 391 for MRE. Scan parameters, including scanner types used at the imaging centre and sequence acquisition parameters, for both LiverMultiScan and MRE are reported in Table S1.

### 2.3 Chronic Liver Disease Subgroups and Referral Specialty

Patients were classified into disease aetiology subgroups according to the diagnosis from their clinical charts at inclusion:

- Steatotic liver disease (SLD): either MASLD, MASH, or metabolic dysfunction and alcohol‐related liver disease (MetALD).
- No diagnosis: no reported diagnosis in clinical charts.
- Other chronic liver disease (Other): any other diagnosis (including haemochromatosis, autoimmune liver disease (AILD), Budd‐Chiari syndrome, viral hepatitis, cirrhosis, etc.).

The management pathways and clinical specialties from which patients were referred to Radiology for routine abdominal MRI were assessed to understand the impact of the technologies on patient standard‐of‐care and treatment outcomes.

### 2.4 Definition of Disease Severity and Worsening

Assessment of disease severity was performed following clinical guideline recommendations [23] and reported thresholds. For identifying the presence of fibrosis, a FIB‐4 threshold of < 1.3 (for those < 65 years) or < 2 (for those ≥ 65 years) was used. In addition, an MRE threshold of < 3.14 kPa (defined as F2 in clinical guidelines) was used to indicate the absence of significant/advanced fibrosis [23]. Overall fibrosis risk was defined using FIB‐4 and MRE thresholds, with those who had a low‐fibrosis risk having both FIB‐4 and MRE under the stated thresholds. For liver disease activity, cT1 values > 800 ms were taken to indicate elevated fibro‐inflammatory disease activity [24], while LFC values ≥ 10% were taken to indicate significant steatosis and LIC ≥ 1.8 mg/g was taken to indicate iron overload.

With the recent approval of therapies to support management of SLD, clinical guidelines have noted disease monitoring to be an area of unmet need. A relative change in both ALT and AST levels of 10% has been shown to predict histological improvement in chronic liver disease [25] and pharmacotherapeutic clinical trials [26]. Thus, in this study, disease improvement was defined as a 10% relative reduction in both ALT and AST. Similarly, increases in ALT and AST levels are indicative of disease worsening [23] with studies showing that even small changes in ALT [27] and AST [28] are predictive of histologic and fibrotic progression in MASLD patients. Moreover, both the Liver Forum [29] and the EASL‐EASD‐EASO guidelines [30] emphasise that no specific ALT or AST thresholds for worsening disease have been established. Thus, any rise in these enzymes should prompt noninvasive fibrosis assessment and closer follow‐up, since increasing transaminases often foreshadow disease progression. In accordance with this, in this study, disease progression (worsening) was defined as a 10% relative increase in both ALT and AST.

### 2.5 Statistical Data Analysis

Variables were reported as either mean and standard deviation, median and range, or frequency and percentage as appropriate.

Due to the recent approval of drug therapy for MASLD and the need to evaluate the utility of NITs for monitoring, subgroup analyses in the low‐fibrosis risk subgroup were performed to explore the added benefit of using MRI techniques in the management of patients with SLD. To support the evaluation of disease worsening or improvement during SLD monitoring, patients were grouped as having a combination of either high or low MRE (fibrosis) and cT1 (disease activity). The Kruskal–Wallis test and Pearson's Chi‐squared test for categorical data were used to compare across the different disease aetiology subgroups.

During the monitoring period making up longitudinal follow‐up, the relative change (increase/decrease) in laboratory biochemical (blood) markers was evaluated. Alongside this evaluation, two‐sample *t*‐tests were used to investigate the difference in worsening versus no worsening or improvement in liver disease. Kaplan–Meier analyses were used to evaluate the probability of disease progression (worsening) in the whole study group over the follow‐up period. The area under the receiver operating characteristic (AUC) was also used to investigate the utility of baseline imaging to predict those with SLD who had disease progression.

## 3 Results

### 3.1 Participant Demographics and Referral Pathways

**FIGURE 1:**
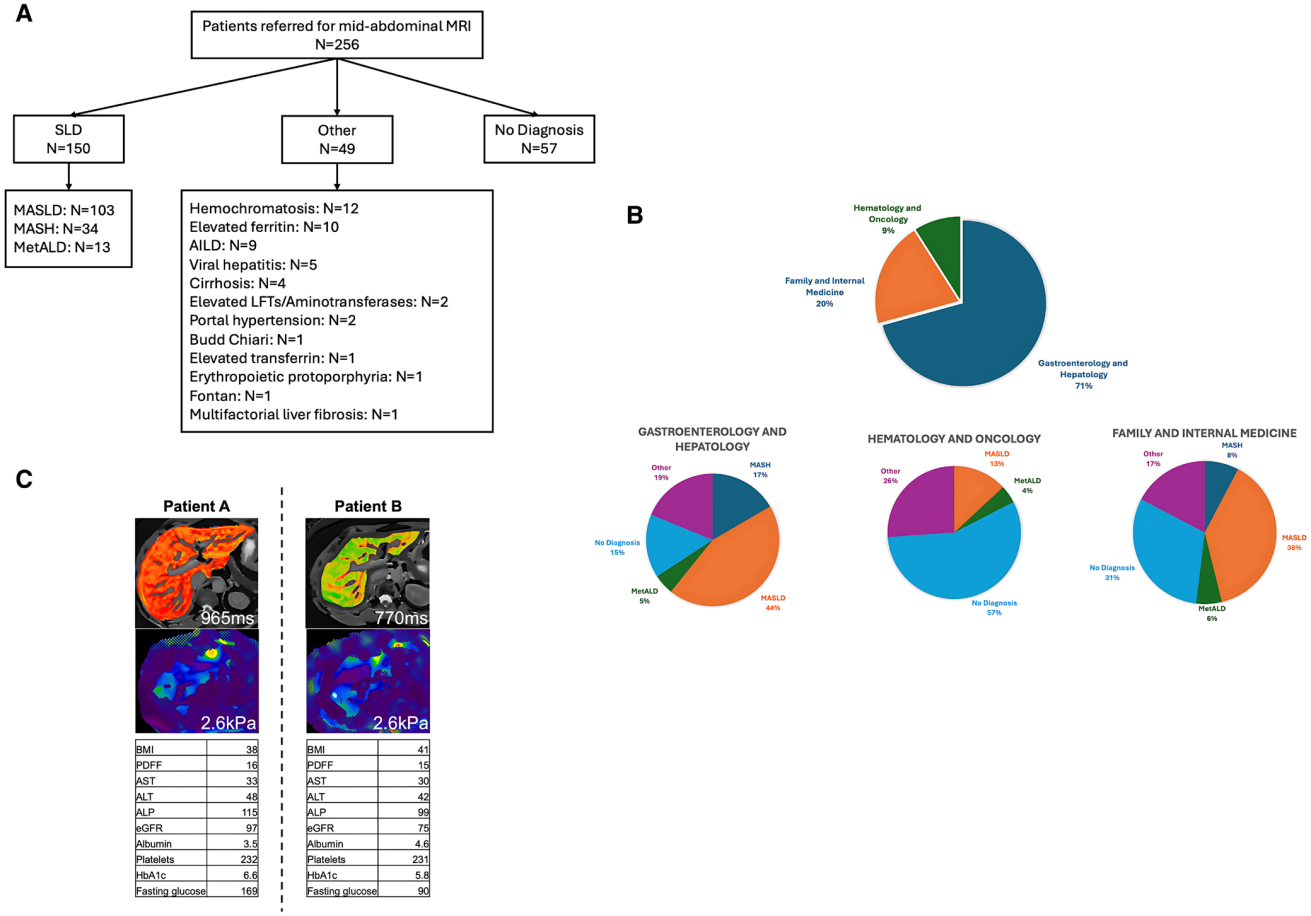
Patient demographics illustrating (A) the patients referred for abdominal magnetic resonance imaging (MRI) scan as part of their standard‐of‐care, and (B) the subspecialty of the referring physician. (C) cT1 and MRE maps for two White female patients with MASLD. Patient A is 63 years old whilst patient B is 75 years old.

Over 18 months, between February 2022 and July 2024, 256 patients with diagnosed or suspected chronic liver disease (CLD) were referred to the Massachusetts General Hospital (MGH) Radiology Department for a non‐contrast abdominal MRI scan as part of their SoC. An average of 14 patients were scanned per month (3–4 patients per week; range: 4–28 per month) for varying liver etiologies across at least 15 ICD‐10 codes (including MASLD, MASH, MetALD, AILD, viral hepatitis and cirrhosis) (Table 1, Figure 1). Patients were aged 19–82 years (mean age: 53 ± 15 years) with 51% being female, 48% having BMI > 30 kg/m^2^ and the majority (85%) White (Caucasian) (Table 1).

**TABLE 1:** Patient demographics, laboratory biochemical (blood) markers, imaging markers and referring physician specialty.

Disease aetiology	SLD (*N* = 150)					Other (*N* = 49)	No diagnosis (*N* = 57)	*p* (SLD vs. other/no diagnosis)	*p* (all groups)
	MASLD (*N* = 103)	MASH (*N* = 34)	MetALD (*N* = 13)	*p*	Total SLD (*N* = 150)				
Age (years)	56.7 (14.9)	53.8 (15.1)	50.2 (8.9)	0.097	55.5 (14.6)	53.8 (16.3)	49.8 (15.1)	0.092	0.061
Sex (%, F)	51 (49.5%)	21 (61.8%)	3 (23.1%)	0.059	75 (50.0%)	33 (67.3%)	23 (40.4%)	0.655	**0.019**
Race/ethnicity				**0.007**				0.308	0.122
White Anglo American (White Caucasian)	88 (85.4%)	22 (64.7%)	11 (84.6%)		121 (80.7%)	46 (93.9%)	44 (77.2%)		
Hispanic	10 (9.7%)	9 (26.5%)	1 (7.7%)		20 (13.3%)	2 (4.1%)	5 (8.8%)		
Black or African American	1 (1.0%)	—	—		1 (0.7%)	—	4 (7.0%)		
Other	2 (1.9%)	2 (5.9%)	—		2 (1.3%)	—	1 (1.8%)		
No response	6 (5.8%)	8 (23.5%)	2 (15.4%)		6 (4.0%)	1 (2.0%)	3 (5.3%)		
BMI (kg/m^2^)	32.9 (7.0)	36.2 (9.4)	31.6 (4.1)	0.313	33.5 (7.5)	29.0 (6.3)	29.3 (7.1)	**< 0.001**	**< 0.001**
Liver markers									
ALT (IU/L)	52.0 (41.4)	54.9 (46.0)	70.0 (55.1)	0.585	54.3 (43.8)	40.6 (31.5)	50.2 (49.3)	**0.041**	0.085
AST (IU/L)	40.2 (22.5)	38.9 (20.7)	65.3 (62.0)	0.447	42.3 (28.7)	36.2 (23.9)	42.3 (32.5)	0.072	0.181
Albumin (g/dL)	4.5 (0.5)	4.5 (0.3)	4.6 (0.5)	0.311	4.5 (0.5)	4.4 (0.4)	4.4 (0.5)	**0.016**	0.055
Platelets (×10^3^)	242.8 (77.5)	257.5 (96.4)	173.3 (49.5)	**0.018**	241.2 (82.4)	227.4 (83.1)	247.5 (111.9)	0.843	0.951
FIB‐4	1.6 (1.0)	1.4 (1.1)	2.7 (1.8)	**0.021**	1.6 (1.1)	1.9 (2.3)	1.3 (0.8)	0.204	0.349
Total bilirubin (mg/dL)	0.6 (0.3)	0.5 (0.2)	0.7 (0.4)	0.402	0.6 (0.3)	0.7 (0.6)	0.6 (0.3)	0.782	0.888
ALP (IU/L)	85.6 (32.8)	99.6 (44.7)	112.7 (41.7)	**0.021**	91.2 (37.4)	99.8 (65.6)	111.8 (119.0)	0.766	0.956
Metabolic markers									
HbA1c (mmol/mol)	6.3 (1.4)	6.3 (1.3)	5.9 (0.9)	0.613	6.3 (1.3)	5.9 (0.6)	5.9 (1.2)	0.115	0.265
Fasting Glucose (mg/dL)	111.3 (43.1)	114.7 (30.5)	114.4 (27.3)	0.462	112.3 (39.5)	101.6 (19.1)	125.4 (79.4)	0.161	0.29
eGFR	91.4 (19.2)	92.9 (17.2)	90.8 (20.6)	0.92	91.6 (18.8)	89.6 (19.4)	91.9 (21.9)	0.728	0.702
Creatinine (mg/dL)	0.9 (0.2)	1.1 (1.2)	1.0 (0.4)	0.117	0.9 (0.6)	1.1 (1.4)	0.8 (0.2)	0.988	0.389
Urea (BUN) (mg/dL)	15.1 (5.3)	12.2 (4.9)	13.9 (5.0)	**0.031**	14.4 (5.3)	15.4 (5.1)	15.6 (6.0)	0.123	0.301
Imaging markers									
cT1 (ms)	818.5 (116.5)	849.6 (96.3)	881.0 (111.0)	**0.049**	831.0 (113.0)	779.7 (117.3)	776.2 (107.7)	**< 0.001**	**< 0.001**
MRE (kPa)	2.8 (1.3)	3.0 (1.1)	3.5 (1.7)	0.08	2.9 (1.3)	3.1 (1.8)	2.5 (0.9)	0.086	**0.025**
PDFF (%)	11.3 (8.7)	13.9 (9.3)	14.3 (10.0)	0.211	12.1 (9.0)	6.5 (7.2)	7.1 (6.7)	**< 0.001**	**< 0.001**
Referring physician									
Gastroenterology and Hepatology	80 (77.7%)	30 (88.2%)	9 (69.2%)		119 (79.3%)	34 (69.4%)	28 (49.1%)		
Family and Internal Medicine	20 (19.4%)	4 (11.8%)	3 (23.1%)		27 (18.0%)	9 (18.4%)	16 (28.1%)		
Haematology and Oncology	3 (2.9%)	—	1 (7.7%)		4 (2.7%)	6 (12.2%)	13 (22.8%)		

*Note:* Bold font was used to indicate *p*‐values indicating statistically significant comparisons.

Of those referred for imaging, 60% had pre‐existing conditions, including type‐2 diabetes mellitus (T2DM) (24%), hyperlipidemia (39%), obesity (31%), dyslipidemia (4%), and hypertension (30%). Regarding the use of recently approved treatments (glucagon‐like peptide‐1 [GLP‐1] and glucagon‐like peptide‐1 receptor agonist [GLP‐RA]), 11% with T2DM were on semaglutide and 6% were on tirzepatide, while 18% of those with obesity were on semaglutide and 6% were on tirzepatide. Regarding smoking, 3% were active smokers, 25% were former smokers, and 71% were non‐smokers. Although the majority, 46%, did not drink any alcohol, 34% consumed ≥ 14 units of alcohol per week, whilst 20% were moderate alcohol consumers (1–13 units per week).

Patients were referred for an MRI as part of their SoC (patient monitoring) or to support diagnosis from three specialty care pathways: Gastroenterology and Hepatology (71%), Haematology and Oncology (20%), and Family and Internal Medicine (9%). The main reason for referral was SLD, which accounted for 66% of patients from the Gastroenterology and Hepatology specialty, 57% from Haematology and Oncology and 52% from Family and Internal Medicine (Table 1, Figure 1). Additionally, 22% of patients did not have a stated diagnosis at the time of referral, while 19% had other mixed chronic liver diseases (Table 1, Figure 1).

Both MRE and LiverMultiScan had good technical performance, with only 6% and 4% failure rates respectively. The main reasons for LiverMultiScan failure were non‐reported cT1 values in patients due to LFC > 35%; in these cases, a reduced report with LFC and LIC was provided. The main reasons for MRE failure were technical failure and elevated liver iron.

### 3.2 Clinical Management and Investigations: Baseline

In addition to imaging, all patients had a blood panel laboratory assessment. Comparisons between the groups showed that age, BMI, MRE, LFC and cT1 were significantly different among the groups (Table 1). Those with MASLD were the oldest, whereas those with no diagnosis were the youngest. Similarly, those with MASH had the highest BMI, while those with no diagnosis had the lowest. Overall, those with MetALD exhibited the worst metabolic health, with elevated ALT and AST levels, FIB‐4 score, cT1, MRE and LFC as well as lowest platelet values compared with all other subgroups (Table 1). Figure 1 shows an illustration of cT1 and MRE for two patients with MASLD.

No biopsies were performed as part of standard‐of‐care for any of the patients evaluated. There were statistically significant associations between blood markers (ALT, AST, Albumin, platelets, FIB‐4) and imaging markers (cT1, MRE, PDFF) (−0.22 ≤ *R* ≤ 0.55, *p* < 0.05) (Figure S1).

**FIGURE 2:**
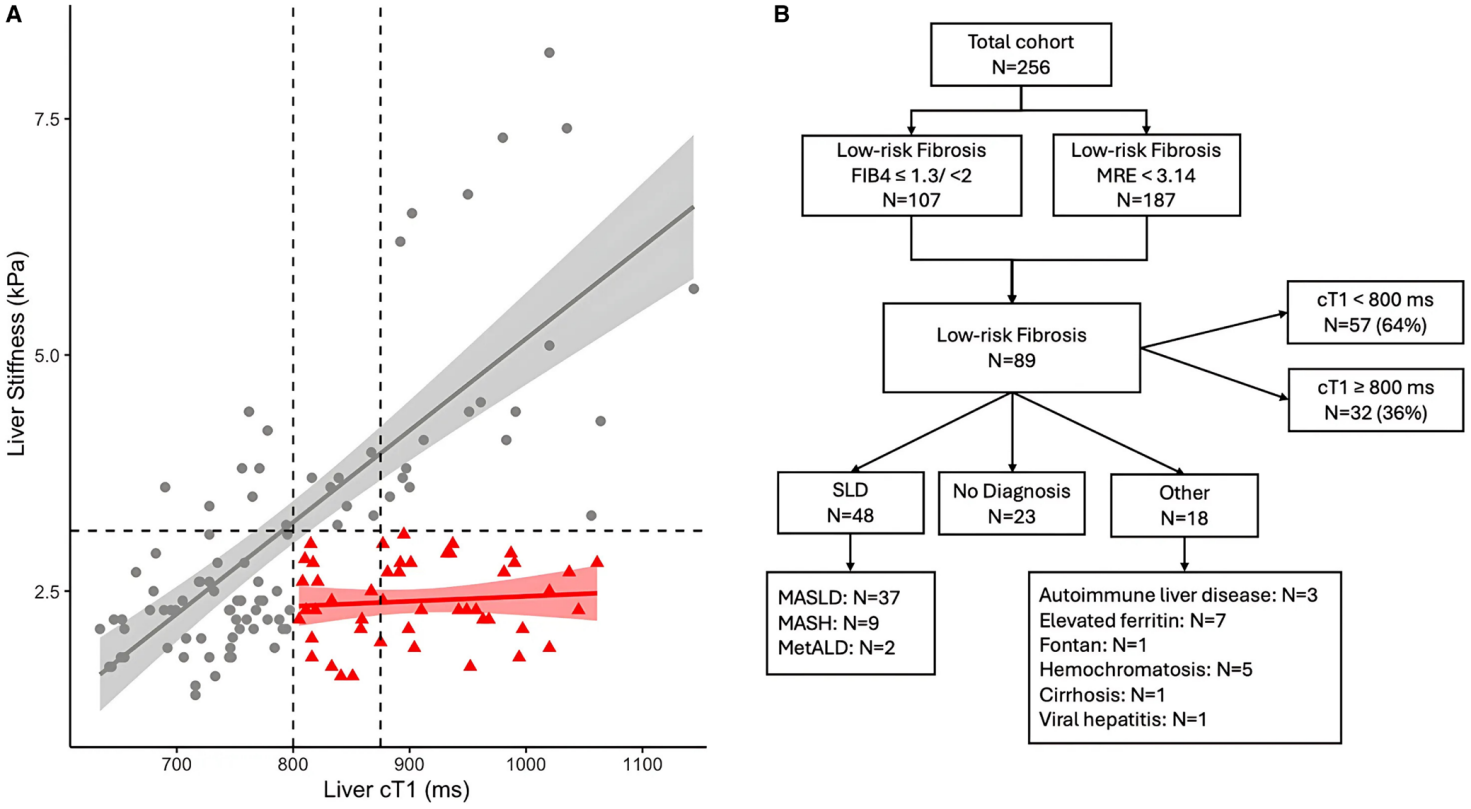
Association between liver disease activity (cT1) and liver fibrosis (MRE). Dashed lines indicated stage F2 fibrosis (MRE: 3.14 kPA), active liver disease (cT1: 800 ms) and high‐risk liver disease (cT1: 875 ms). (B) Proportion of the patient population with a low fibrosis risk showing the disease aetiology and distribution of cT1 values in the patients with a low fibrosis risk.

Due to the limitations of FIB‐4 and poor sensitivity of ultrasound‐based technologies (such as VCTE), MRE is typically preferred as a fibrosis marker at MGH. Because cT1 and MRE provide information on complementary aspects of disease, that is, disease activity and advanced fibrosis respectively, we investigated further the relationship between these two markers. Figure 2 shows the relationship between cT1 and MRE. Most participants (73%) had low MRE (< 3.14 kPa, ≤ F2) and 42% had low FIB‐4 (42%), with 36% having elevated cT1 (Figure 2). Of those with both low MRE and low FIB‐4 results (35%), most had SLD (54%, 48/89), with the remaining having either no diagnosis (26%, 23/89) or another CLD (20%, 18/89) (Figure 2). All patients with elevated MRE indicative of cirrhosis (MRE > 5 kPa) also had elevated cT1 (> 875 ms).

In the low‐fibrosis risk subgroup, despite having similar age, BMI and laboratory biochemical markers (including FIB‐4), those with SLD had significantly higher cT1 and PDFF (Table 2). Although the majority had low cT1 (< 800 ms), the 36% (32/89) with elevated cT1 were younger with higher BMI and a significantly higher HbA1c, urea and estimated glomerular filtration rate (eGFR) (Table 3).

**TABLE 2:** Patient demographics for individuals within low‐fibrosis risk subgroup. Low‐fibrosis risk was defined as.

	SLD (*N* = 48)			No diagnosis (*N* = 23)	Other (*N* = 18)	*p*
	MASLD (*N* = 37)	MASH (*N* = 9)	MetALD (*N* = 2)			
Age (years)	49.0 (14.4)	47.2 (15.4)	51.0 (1.4)	48.2 (15.3)	48.6 (16.3)	1
BMI (kg/m^2^)	32.3 (7.6)	34.4 (6.3)	31.8 (2.8)	30.6 (9.1)	30.2 (5.8)	0.678
Laboratory blood markers						
ALT (IU/L)	42.5 (25.5)	55.9 (49.2)	33.5 (2.1)	40.3 (53.6)	36.9 (23.6)	0.677
AST (IU/L)	28.6 (11.0)	34.6 (21.6)	31.0 (1.4)	31.7 (28.0)	25.6 (8.3)	0.743
Albumin (g/dL)	4.5 (0.7)	4.5 (0.4)	4.8 (0.1)	4.4 (0.4)	4.4 (0.3)	0.243
Platelets (×10^3^)	286.7 (75.3)	295.6 (84.0)	222.5 (3.5)	277.7 (99.2)	260.8 (69.7)	0.358
FIB‐4	0.8 (0.3)	0.8 (0.3)	1.2 (0.1)	0.9 (0.3)	0.9 (0.4)	0.274
Total bilirubin (mg/dL)	0.6 (0.4)	0.5 (0.3)	0.8 (0.3)	0.5 (0.3)	0.5 (0.2)	0.407
ALP (IU/L)	79.1 (25.2)	87.3 (29.2)	123.0 (70.7)	88.1 (40.1)	106.3 (87.8)	0.705
HbA1c (mmol/mol)	6.0 (1.1)	5.9 (0.3)	5.5 (0.4)	5.7 (1.0)	5.8 (0.5)	0.665
Fasting glucose (mg/dL)	104.1 (25.4)	122.1 (40.9)	103.0 (19.8)	107.4 (36.1)	105.9 (23.7)	0.758
eGFR	96.0 (17.9)	96.6 (13.2)	80.5 (33.2)	94.6 (25.6)	92.6 (17.0)	0.805
Urea (BUN) (mg/dL)	13.9 (4.3)	10.3 (4.5)	17.0 (8.5)	15.6 (7.7)	15.3 (4.9)	0.148
Imaging markers						
cT1 (ms)	806.8 (108.4)	839.6 (57.6)	806.0 (100.4)	774.7 (98.6)	734.6 (103.7)	**0.039**
MRE (kPa)	2.2 (0.4)	2.4 (0.5)	2.9 (0.1)	2.2 (0.3)	2.2 (0.3)	0.194
PDFF (%)	11.5 (9.2)	11.6 (5.8)	9.5 (6.4)	6.3 (5.7)	8.0 (8.3)	**0.016**

*Note:* Bold font was used to indicate *p*‐values indicating statistically significant comparisons.

**TABLE 3:** Referring specialist and distribution of laboratory biochemical (blood) markers of liver, metabolic health, and imaging markers for those with low fibrosis risk (FIB‐4 < 1.3/< 2 and MRE < 3,14 kPa).

Low fibrosis risk	cT1 > 800 ms (*N* = 32)	cT1 < 800 ms (*N* = 57)	*p*
Age (years)	47.6 (14.3)	49.0 (15.3)	0.683
BMI (kg/m^2^)	33.0 (8.1)	30.9 (7.3)	0.189
Sex (%, F)	22 (68.8%)	30 (56.6%)	0.266
Liver markers			
ALT (IU/L)	52.4 (51.4)	35.2 (23.6)	0.067
AST (IU/L)	33.1 (23.8)	27.5 (13.0)	0.178
Albumin (g/dL)	4.5 (0.4)	4.4 (0.6)	0.642
Platelets (×10^3^)	281.7 (70.6)	275.3 (88.4)	0.404
FIB‐4	0.8 (0.3)	0.9 (0.3)	0.441
Total bilirubin (mg/dL)	0.5 (0.3)	0.5 (0.3)	0.335
ALP (IU/L)	91.1 (33.3)	86.2 (57.9)	0.081
Metabolic markers			
HbA1c (mmol/mol)	6.0 (1.0)	5.7 (0.9)	**0.045**
Fasting glucose (mg/dL)	109.3 (38.0)	104.0 (20.5)	0.901
Urea (BUN) (mg/dL)	13.0 (6.9)	15.2 (5.0)	**0.004**
Creatinine (mg/dL)	1.3 (2.0)	0.9 (0.2)	0.201
eGFR	98.3 (22.5)	91.9 (18.4)	**0.04**
Imaging markers			
MRE (kPa)	2.3 (0.4)	2.2 (0.3)	0.127
PDFF (%)	12.7 (7.3)	5.7 (3.5)	**< 0.001**
Diagnosis			
No diagnosis	8 (25.0%)	15 (28.3%)	0.152
Other	3 (9.4%)	13 (24.5%)	
SLD	21 (65.6%)	25 (47.2%)	
Referring physician			
Family and Internal Medicine	7 (21.9%)	11 (20.8%)	0.599
Gastroenterology and Hepatology	23 (71.9%)	35 (66.0%)	
Haematology and Oncology	2 (6.2%)	7 (13.2%)	

*Note:* Bold font was used to indicate *p*‐values indicating statistically significant comparisons.

### 3.3 Clinical Management and Investigations: Longitudinal Management and Follow‐Up

After 6–12 months (range: 100–777 days), depending on their aetiology, most patients (79%, 201/256) received follow‐up monitoring; of this group, most had SLD (56%, 112/201). At follow up, only laboratory biochemical markers were evaluated; no liver biopsies or imaging were performed. Depending on the disease aetiology (56% SLD, 24% no diagnosis, 20% other), patients were managed according to SoC as determined by their treating practitioner. Compared with their counterparts who had a lower risk of fibrosis, those with advanced fibrosis were managed more closely and recommended for repeated LFTs every 3–6 or 6–12 months, with specific goals of decreasing liver fat and, where applicable, alcohol consumption. A proportion of patients (10%) with elevated BMI levels and liver blood tests were referred to the MGH Weight Centre (MGH‐WC) for consideration of bariatric surgery.

In addition to evaluating the changes in their metabolic and liver biochemical profiles over the follow‐up period, and to further enhance understanding of their risk profile, patients with either elevated cT1 or MRE (or both) (≥ 800 ms and ≥ 3.14 kPa, respectively) were compared with those who had low‐risk disease (cT1 < 800 ms and MRE < 3.14 kPa). Most patients (57%, 115/201) had low fibrosis risk and low disease activity, while 20% (41/201) had high‐risk disease (both elevated MRE and cT1) (Table 4). Of those with a combination of high and low MRE and cT1 values, the minority (6%, 13/201) had elevated fibrosis (MRE > 3.14 kPa) with low disease activity (cT1 < 800 ms), while the remaining (16%, 32/201) had low fibrosis risk with elevated disease activity (cT1 > 800 ms) (Table 4).

**TABLE 4:** Relative changes (%) in laboratory biochemical (blood) markers over across subgroups defined by cT1 and MRE. Low MRE was defined as < 3.14 kPa and low cT1 was defined as < 800 ms. All changes shown are relative to the first visit.

	Low MRE, low cT1 (*N* = 115)	Low MRE, high cT1 (*N* = 32)	High MRE, high cT1 (*N* = 41)	High MRE, low cT1 (*N* = 13)
ALT (%)	−5.9 (50.8)	81.5 (354.3)	−24.5 (42.4)	−19.6 (29.6)
AST (%)	−4.8 (38.6)	51.7 (166.9)	−18.1 (44.8)	−17.8 (25.5)
Albumin (%)	14.4 (126.5)	−1.0 (6.4)	−0.4 (11.2)	−0.3 (5.2)
Platelets (%)	8.6 (56.8)	−3.9 (12.3)	0.1 (25.2)	−5.2 (7.1)
FIB4 (%)	5.8 (74.2)	23.3 (43.7)	−5.3 (36.5)	−4.5 (21.3)
Total bilirubin (%)	21.4 (71.7)	11.2 (69.6)	2.6 (41.6)	13.8 (26.0)
ALP (%)	3.5 (51.2)	3.2 (30.0)	−4.8 (32.1)	−6.4 (4.5)
Urea (BUN) (%)	12.1 (67.7)	75.0 (291.0)	12.2 (71.8)	−2.6 (37.0)
HbA1c (%)	50.4 (213.1)	4.0 (19.5)	3.6 (11.8)	−9.6 (19.9)
Fasting glucose (%)	−2.7 (24.2)	12.6 (42.6)	3.0 (24.0)	−12.1 (28.9)
Baseline imaging markers				
cT1 (ms)	718.1 (46.5)	899.7 (62.4)	928.2 (89.4)	739.1 (43.0)
MRE (kPa)	2.2 (0.4)	2.3 (0.4)	4.8 (1.7)	4.3 (1.7)
PDFF (%)	5.5 (4.1)	12.7 (7.3)	10.9 (8.1)	6.2 (4.7)
Diagnosis				
MASH	8 (7.0%)	6 (18.8%)	7 (17.1%)	2 (15.4%)
MASLD	46 (40.0%)	14 (43.8%)	15 (36.6%)	7 (53.8%)
MetALD	2 (1.7%)	1 (3.1%)	4 (9.8%)	0 (0%)
No diagnosis	33 (28.7%)	8 (25.0%)	5 (12.2%)	2 (15.4%)
Other	26 (22.6%)	3 (9.4%)	10 (24.4%)	2 (15.4%)

**FIGURE 3:**
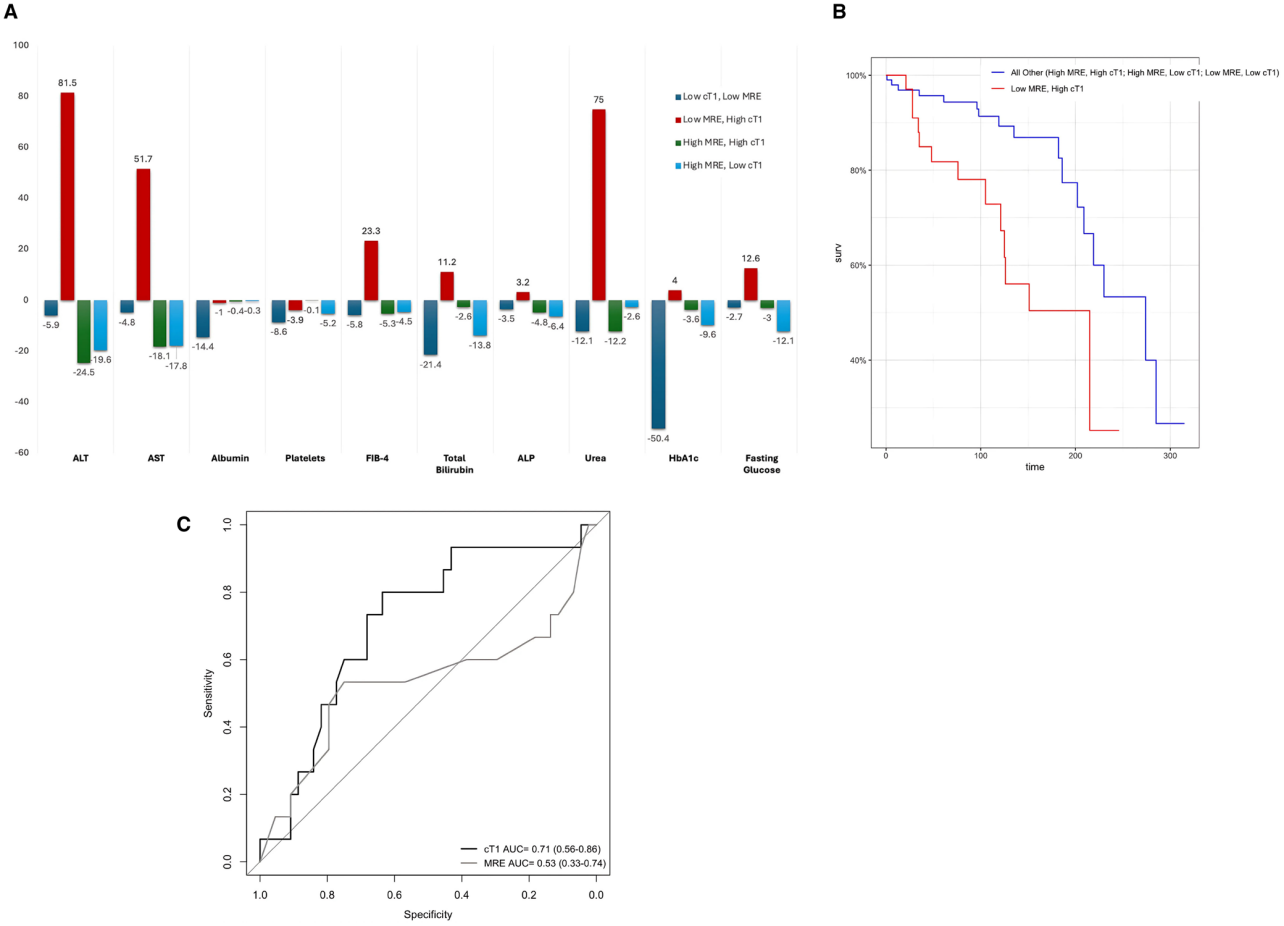
(A) Longitudinal changes on follow up in laboratory biochemical (blood) markers across subgroups defined by cT1 and MRE, and (B) Kaplan–Meyer curves comparing the risk of disease worsening between those with a low fibrosis risk (MRE < 3.14 kPA) and elevated disease activity (cT1). (C) Performance of cT1 and MRE to predict SLD participants who had a worsening in their liver disease over a 12‐month follow‐up period.

Those with both low disease activity (cT1 < 800 ms), and fibrosis (MRE < 3.14 kPa) had improvement in all markers over the follow‐up period, regardless of disease aetiology (Table 4). Similarly, following the closer and stricter monitoring, those with elevated MRE (regardless of cT1 elevation) also had an improvement in their blood markers (both liver and metabolic function markers) (Table 4). However, compared with all other patients, those with low fibrosis risk (MRE < 3.14 kPa) but elevated liver disease activity (cT1 > 800 ms) were the only subgroup of patients to have a worsening in their disease across all blood markers (Tables 4 and 5, Figure 3).

**TABLE 5:** Comparisons between those who had worsening vs. no‐worsening/improvement in their liver disease during the follow‐up period. Disease worsening was defined as 10% relative reduction in both ALT and AST.

	No worsening/improvement (*N* = 159)	Worsening (*N* = 42)	*p*
Age (years)	54.8 (14.8)	51.5 (15.7)	0.276
BMI (kg/m^2^)	31.3 (8.1)	30.0 (7.0)	0.562
Sex (%, F)	89 (55.9%)	24 (58.1%)	0.831
Liver markers			
ALT (IU/L)	57.3 (46.6)	34.9 (17.5)	**0.012**
AST (IU/L)	47.7 (34.3)	28.9 (11.9)	**0.001**
Albumin (g/dL)	4.4 (0.5)	4.3 (0.4)	0.171
Platelets (×10^3^)	238.8 (84.1)	242.0 (121.5)	0.841
FIB‐4	1.8 (1.4)	1.5 (2.0)	**0.029**
Total bilirubin (mg/dL)	0.7 (0.5)	0.6 (0.3)	0.622
ALP (IU/L)	101.0 (54.2)	83.4 (26.8)	0.122
Metabolic markers			
Fasting glucose (mg/dL)	114.1 (55.0)	120.4 (61.4)	0.506
HbA1c (mmol/mol)	6.1 (1.3)	6.0 (0.7)	0.528
eGFR	91.4 (19.2)	88.3 (27.5)	0.927
Creatinine (mg/dL)	0.9 (0.2)	1.4 (2.0)	0.539
Urea (BUN) (mg/dL)	14.8 (5.0)	14.5 (7.4)	0.302
Imaging markers			
cT1 (ms)	805.0 (115.5)	844.6 (113.9)	**0.047**
MRE (kPa)	2.9 (1.5)	2.9 (1.3)	0.331
PDFF (%)	9.8 (9.1)	8.9 (6.6)	0.732
Diagnosis			
SLD	90 (56.8%)	26 (61.3%)	0.901
No diagnosis	38 (23.7%)	7 (16.1%)	
Other CLD	31 (19.5%)	9 (22.6%)	

*Note:* Bold font was used to indicate *p*‐values indicating statistically significant comparisons.

### 3.4 SLD Subgroup Management

All SLD patients received diet and lifestyle recommendations made for weight loss, with alcohol avoidance recommended for those with MetALD. To evaluate disease progression with regards to baseline imaging markers, patients were sub‐categorised using cT1 and MRE. Those with SLD who were classified as having low‐risk disease on both cT1 (< 800 ms) and MRE (< 3.14 kPa) experienced improvement in all their markers over the follow‐up period (Table 4, Figure 3). Similarly, those with high MRE but low cT1 experienced improvement across most of their biochemical markers following a period of ~12 months of monitoring. High‐risk patients (cT1 > 800 ms and MRE > 3.14 kPa) had improvement in their liver blood markers albeit with slight worsening in their metabolic markers (HbA1c, fasting glucose, urea, creatinine) (Table 4, Figure 3). Conversely, over the follow‐up period, those with low MRE (< 3.14 kPa) but elevated cT1 (> 800 ms) had consistent worsening in AST, ALT, FIB‐4, fasting glucose and urea (Table 4, Figure 3).

### 3.5 No Diagnosis Subgroup Management

In this study, 22% of those included did not have a diagnosis at baseline. The majority (49%) came from the Gastroenterology and Hepatology specialist pathway, followed by 28% from Family and Internal Medicine and 23% from Haematology and Oncology. In this population, 33% had elevated disease activity (cT1 ≥ 800 ms), 12% had significant fibrosis (MRE ≥ 3.14 kPa) and 26% had high liver fat (PDFF ≥ 10%). These patients were referred for imaging using LiverMultiScan and MRE to support diagnosis.

At follow‐up, 58% (33/57) of those without a diagnosis at baseline had received a diagnosis (23% SLD, 35% Other). However, 42% (24/57) still did not have a stated diagnosis in their clinical notes by the end of the follow‐up period (Figure S2). Regarding their biochemical profile at follow‐up, those without a diagnosis at follow‐up had worsening of their FIB‐4, bilirubin and HbA1c (Figure S3).

### 3.6 Prognostic Utility of NITs: Predicting Worsening Metabolic Profile

Investigations into the prognostic utility of baseline imaging markers to predict disease progression (10% relative increase in both ALT and AST over the follow‐up period); Kaplan–Meier analyses showed that individuals with a low fibrosis risk (MRE < 3.14kPA), but active disease (cT1 > 800 ms) had a significantly higher risk of disease progression/worsening (HR: 3.1 [1.4–6.5], *p* = 0.0035) compared with all other subgroup combinations of MRE and cT1 (Figure 3). Furthermore, in the SLD subgroup (the largest chronic liver disease group being referred for imaging), cT1 was the best performing univariate imaging NIT (AUC: 0.71) and outperformed both MRI‐PDFF (AUC: 0.64) and MRE (AUC: 0.53) for prediction of disease progression/worsening (Figure 3).

## 4 Discussion

In this study on clinical profile, referral trends, and real‐world application of NITs in chronic liver disease management, three main findings were revealed. First, multiparametric imaging has an important role in the longitudinal management of chronic liver disease. Patients with low fibrosis (MRE) but elevated disease activity (corrected T1) have a significantly high risk (HR: 3.1) of disease progression/worsening over 12 months. In SLD, the largest disease group, cT1 outperformed MRE in predicting worsening disease. Second, despite their limitations, blood marker panels remain the primary tool for longitudinal management, while liver biopsy is rarely used in real‐world practice. Third, Gastroenterology and Hepatology specialists are the leading referrers of MRI‐based NITs as part of routine clinical care.

Our study examined patients referred to Radiology for imaging in the management of chronic liver disease. While referrals came from various specialties, Gastroenterology and Hepatology were the primary sources. These fields have seen significant research investment in noninvasive tests (NITs), leading to strong clinical evidence and practice guidelines recommending their use, particularly to reduce reliance on liver biopsy [31, 32]. The importance of NITs is underscored by the global rise of MASLD, which is closely linked to cardiovascular disease, the world's leading cause of death [33, 34]. As these conditions become more prevalent, NITs provide accessible, efficient, and noninvasive diagnostic options. Imaging‐based NITs are increasingly integrated into standard care, with studies showing the growing use of AI‐driven liver MRI solutions that enable scalable adoption without added hardware costs [35].

Despite missing some high‐risk patients [36], as also evidenced in this study, blood tests are traditionally used to monitor patients due to their affordability and accessibility. In contrast, NITs can be repeated serially with minimal risk to the patient, unlike liver biopsy, and offer strong performance for assessing liver disease, making them ideal for longitudinal monitoring [5]. Both LiverMultiScan (CPT: 0648T, 0649T) and MRE (CPT: 76391) are currently being used widely in the United States as part of SoC. Elevated MRE values have been associated with the development of adverse outcomes such as cirrhosis, hepatic decompensation, hepatocellular carcinoma, and mortality [32, 37], while elevated cT1 values have been associated with hepatic decompensation, the need for liver transplantation, disease relapse, hepatocellular carcinoma, cardiovascular events, and mortality [2, 38, 39, 40]. Of those referred to Radiology, the majority (73%) had low‐risk MRE (≤ F2); however, 36% had elevated cT1 (> 800 ms), indicative of increased disease activity and at increased risk of disease progression.

While MRE has well‐known advantages and is recommended for detecting advanced fibrosis and cirrhosis, evidence on its ability to track long‐term changes in early‐stage disease is limited. This is primarily driven by a lack of evidence highlighting how serial results impact clinical management, reproducibility concerns and the lack of method standardisation [41, 42]. Furthermore, as the significance of small liver stiffness fluctuations over time is unclear, the use of MRE for longitudinal monitoring is challenging [43]. MRE is also unsuitable for some conditions like haemochromatosis, which affected 7% of our study population and complicated result interpretation [44]. In our study, only patients with low fibrosis risk but elevated cT1 showed disease progression over 12 months, with a threefold higher risk compared to others.

Population studies show similar cumulative all‐cause mortality rates for both fibrotic and nonfibrotic MASLD [45]. During the monitoring period, 30% of SLD patients with a low fibrosis risk experienced disease progression. From a management perspective, patients with MRE values consistent with advanced fibrosis are already being managed more aggressively with more frequent blood assessments in addition to stringent lifestyle recommendations. Our findings show that regardless of fibrosis risk by MRE, those with elevated cT1 values should be managed as aggressively as those with advanced fibrosis, since they are at risk of disease progression and adverse outcomes [45]. This is particularly important as large studies, both in the general population and in those with MASLD, have shown that elevated cT1 is associated with elevated risk of cardiovascular and liver events [46]. These findings are particularly pertinent because of uncertainty regarding the management of patients with discordant results on imaging. Furthermore, with newly approved MASH therapies, proper treatment selection and monitoring are critical. Currently, cT1 is being used in routine care to assign resmetirom [19]. In this cohort, only 12% were on semaglutide or tirzepatide. Given their risk, patients with low fibrosis scores but elevated cT1 should also be considered for these treatments.

Compared to liver biopsy, NITs offer accurate and repeatable serial markers for managing chronic liver disease. Improvements (decreases) in cT1 have been shown to (1) assess treatment response across different drug mechanisms of action in MASH [47]; (2) monitor MASH improvements following bariatric surgery or dietary changes [48, 49, 50, 51] and (3) evaluate future liver remnant quality after dual‐vein embolization [52] and support liver biopsy avoidance following transplantation [51]. Regarding liver fat, despite not being a prognostic marker of adverse outcomes, reductions in PDFF correlate with improved liver and metabolic health and higher chances of MASH resolution [47, 53]. However, not all markers are suitable for longitudinal follow‐up. While fibrosis markers such as VCTE and FIB‐4 show comparable performance, VCTE has low repeatability, requires different thresholds to identify advanced fibrosis, and is influenced by multiple confounders [10, 12]. Likewise, FIB‐4, despite being an accessible screening tool, is confounded by age and medications (including glucagon‐like peptide‐1 agonists) and misses high‐risk patients with hepatocellular carcinoma [54]. Moreover, in a MASLD population, FIB‐4 has been shown to miss ~60% of patients, mainly women and those with type 2 diabetes under the age of 65 years, with active liver disease [20]. Therefore, MRE remains the most accurate for advanced fibrosis and cirrhosis [32, 55]. Thus, as patient management relies more on the use of NITs, as supported also by regulatory bodies such as FDA [56], combining NITs (primarily imaging‐based NILDAs) to support patient management will be the most viable strategy to avoid adverse outcomes.

Regarding the practical integration of cT1 into existing diagnostic pathways, depending on the setting, clinical guidelines have noted its utility alongside blood markers such as FIB‐4 and elastography imaging markers (VCTE, MRE) [23, 55, 57, 58, 59]. For instance, for patients who fall into indeterminate zones or present with risk factors like obesity or type 2 diabetes, cT1 can be used as a second‐line investigation [60, 61]. When looking at the MASLD diagnostic and management pathway in particular, integration of cT1 earlier in the clinical pathway, alongside blood markers, could support more cost‐effective diagnosis [22] which can support identifying patients missed by FIB‐4 [20], identification of patients eligible for treatment [19], and provide prognostic information related to cardiovascular event risk [46]. In patients with other chronic liver diseases, such as autoimmune liver disease where liver function tests such as ALT and AST are used but can be confounded by medications can impact values, cT1 can be used as a virtual biopsy [62] to inform risk stratification of patients and to assist in the decision to withdraw treatment without the need for repeat liver biopsies [63].

This study had several strengths and some limitations. First, by presenting an assessment of noninvasive tests within real‐world clinical practice at a large academic centre, we highlight the current SoC and incorporation of imaging NITs across a range of chronic liver diseases. Second, by incorporating multiple disease types, the study demonstrates the broad applicability of NITs (primarily imaging‐based NILDAs), showing their utility in monitoring and managing patients in a disease‐agnostic way. Third, by evaluating the current use of NITs in tandem, we highlight the additional clinical information provided by LiverMultiScan, over and above that provided by MRE, that can be used to guide management pathways for patients with elevated disease activity (cT1 > 800 ms) but a low fibrosis risk (MRE < 3.14 kPa). It is worth noting, however, if being considered for large scale screening programmes, when compared to LiverMultiScan, MRE is affected by motion [64], has a high repeatability coefficient across different scanner types and field strengths (22% [41] compared to 1.7% for LiverMultiScan [15]), requires skilled onsite personnel and additional hardware, and is affected by several contraindications (such as inflammation and iron overload [64]). Moreover, although cT1 has shown clinical utility in the assessment of alternative causes of liver inflammation such as viral hepatitis [40], liver congestion (e.g., from tricuspid regurgitation or Fontan's circulation [65]), in this study we did not adjust for potential confounders, such as medication effects, which may influence cT1 values. On the other hand, the absence of follow‐up imaging NIT monitoring means the study relies heavily on blood tests, which may not capture disease progression as effectively. Additionally, the reference standard for diagnosis, liver biopsy, was not performed at this centre as part of SoC. Although work has already been done evaluating the cost‐effectiveness of using MRI‐based NITs in routine clinical practice, future work should focus specifically on exploring how these NITs may help to avoid downstream costs associated with disease progression, complications, and the management of adverse outcomes in patients with chronic liver disease.

In conclusion, we aimed to investigate the use of NITs in routine clinical practice in a large academic medical centre with the objective of understanding the clinical profile, referral trends, and real‐world application to support management of patients with chronic liver diseases referred for imaging. Our findings showed that, regardless of disease aetiology, in addition to providing complementary information, patients with a low fibrosis risk (assessed using MRE) but with elevated disease activity (assessed using cT1) are at a high risk of disease progression. Incorporating both biomarkers as part of SoC to detect disease progression, especially for those with MASLD, is feasible and identifies patients who may benefit from changes in their management.

## Author Contributions

Conceptualization: M.H. Methodology: M.H., K.E.C. Data collection and curation: N.N. Formal analysis: M.H., N.N. Writing – original draft: M.H., N.N. Writing – review and editing: K.E.C., R.T.C., E.D.B., J.E.S., K.L.A., I.B., L.S.F., A.R.K., L.E.D., N.N., M.H. All authors contributed and approved the final manuscript.

## Conflicts of Interest

Mukesh Harisinghani is a consultant for Perspectum. Kathleen E. Corey has received grant support from Novartis, BMS, Boehringher‐Ingelheim. Raymond T. Chung has received grant support from Abbvie, Gilead, Merck, Janssen, Bristol‐Myers Squibb, Roche and Boehringer Ingelheim. Lawrence Friedman receives financial support from Newton‐Wellesley Hospital and receives royalties from Elsevier, McGraw‐Hill and Wiley. Laura E. Dichtel has received study medication from Pfizer, research support from Perspectum Ltd., Lumos Pharma, Recordati and Novo Nordisk, has equity in Marea Therapeutics and Merida Biosciences, and is a consultant for Lumos Pharma, Novo Nordisk and Flare Therapeutics. All other clinicians have no conflicts of interest relevant to this work to disclose.

## Supporting information


**Figure S1:** Correlation plot illustrating the associations between laboratory biochemical (blood) markers and imaging markers. Ellipse area reflect the absolute value of the corresponding Spearman's correlation coefficient, and their eccentricity is parametrically scaled to the correlation value. Only significant associations are indicated.

**Figure S2:** Distribution of patients without diagnosis at baseline and the changes in diagnosis over the follow‐up period.

**Figure S3:** Longitudinal changes in laboratory biochemical (blood) markers across subgroups defined by cT1 and MRE for those without a diagnosis at both baseline and follow‐up.

**Table S1:** Scanner types, scanner parameters and sequence acquisition parameters for LiverMultiScan and MRE.

## Data Availability

The data and analytic methods used in this study remain the property of the individual study sponsors. All deidentified participant data may be made available to other researchers on request following permission, investigator support and following a signed data access agreement.
