# Supplementary material for: Real‐World Assessment of Liver Corrected T1 and Magnetic Resonance Elastography in Predicting Liver Disease Progression
Source: Liver Int. 2025 Aug 14;45(9):e70280. doi: 10.1111/liv.70280 (PMC12351529; doi:10.1111/liv.70280)

**Supplementary Figure 1**: Correlation plot illustrating the associations between laboratory biochemical (blood) markers and imaging markers. Ellipse area reflect the absolute value of the corresponding Spearman’s correlation coefficient, and their eccentricity is parametrically scaled to the correlation value. Only significant associations are indicated.


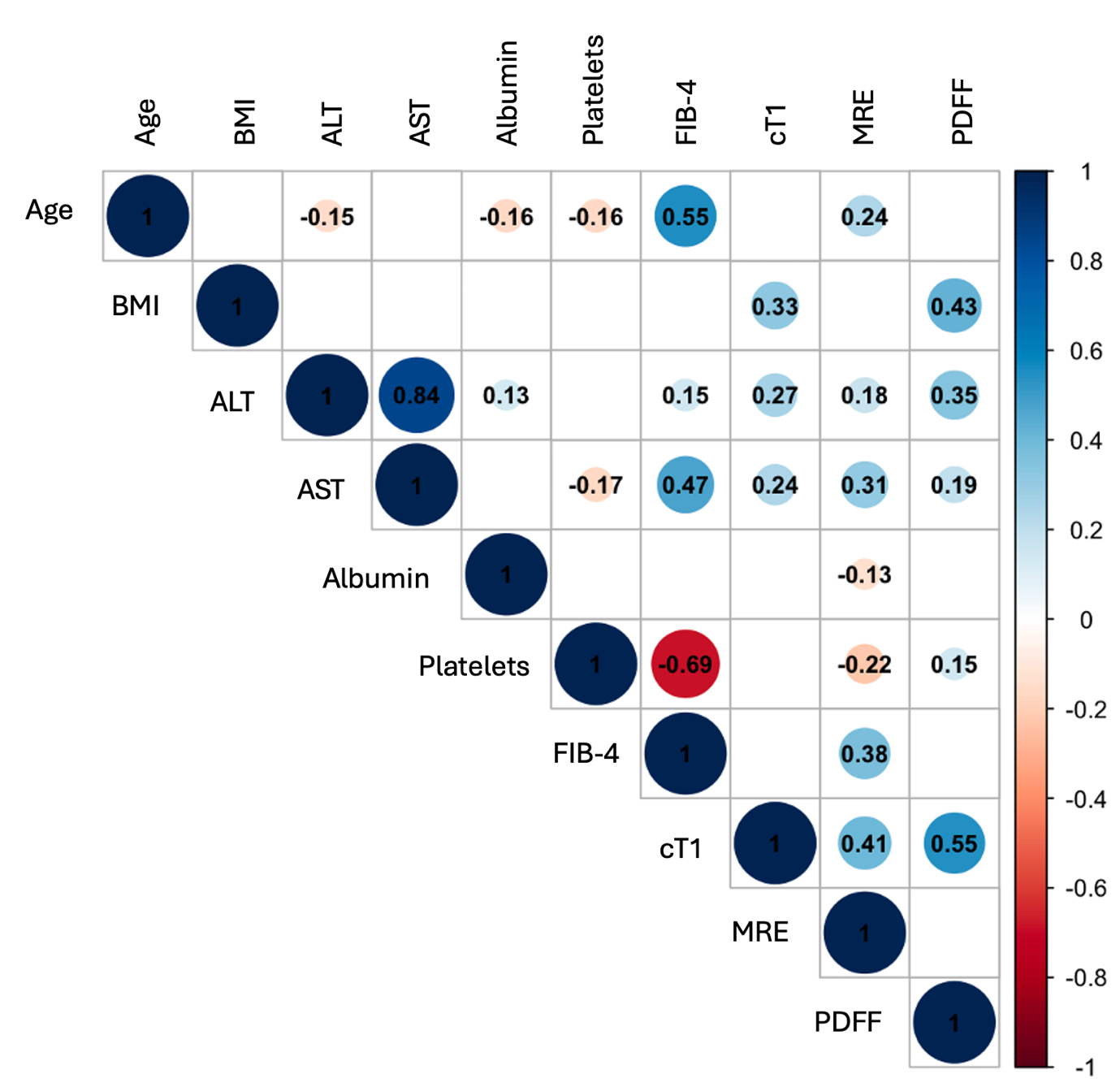

Supplement: Supplementary file 1 — Figure S1: Correlation plot illustrating the associations between laboratory biochemical (blood) markers and imaging markers. Ellipse area reflect the absolute value of the corresponding Spearman's correlation coefficient, and their eccentricity is parametrically scaled to the correlation value. Only significant associations are indicated. [file LIV-45-0-s004.docx]
