# Supplementary material for: Real‐World Assessment of Liver Corrected T1 and Magnetic Resonance Elastography in Predicting Liver Disease Progression
Source: Liver Int. 2025 Aug 14;45(9):e70280. doi: 10.1111/liv.70280 (PMC12351529; doi:10.1111/liv.70280)

**Supplementary Figure 2**: Distribution of patients without diagnosis at baseline and the changes in diagnosis over the follow-up period.


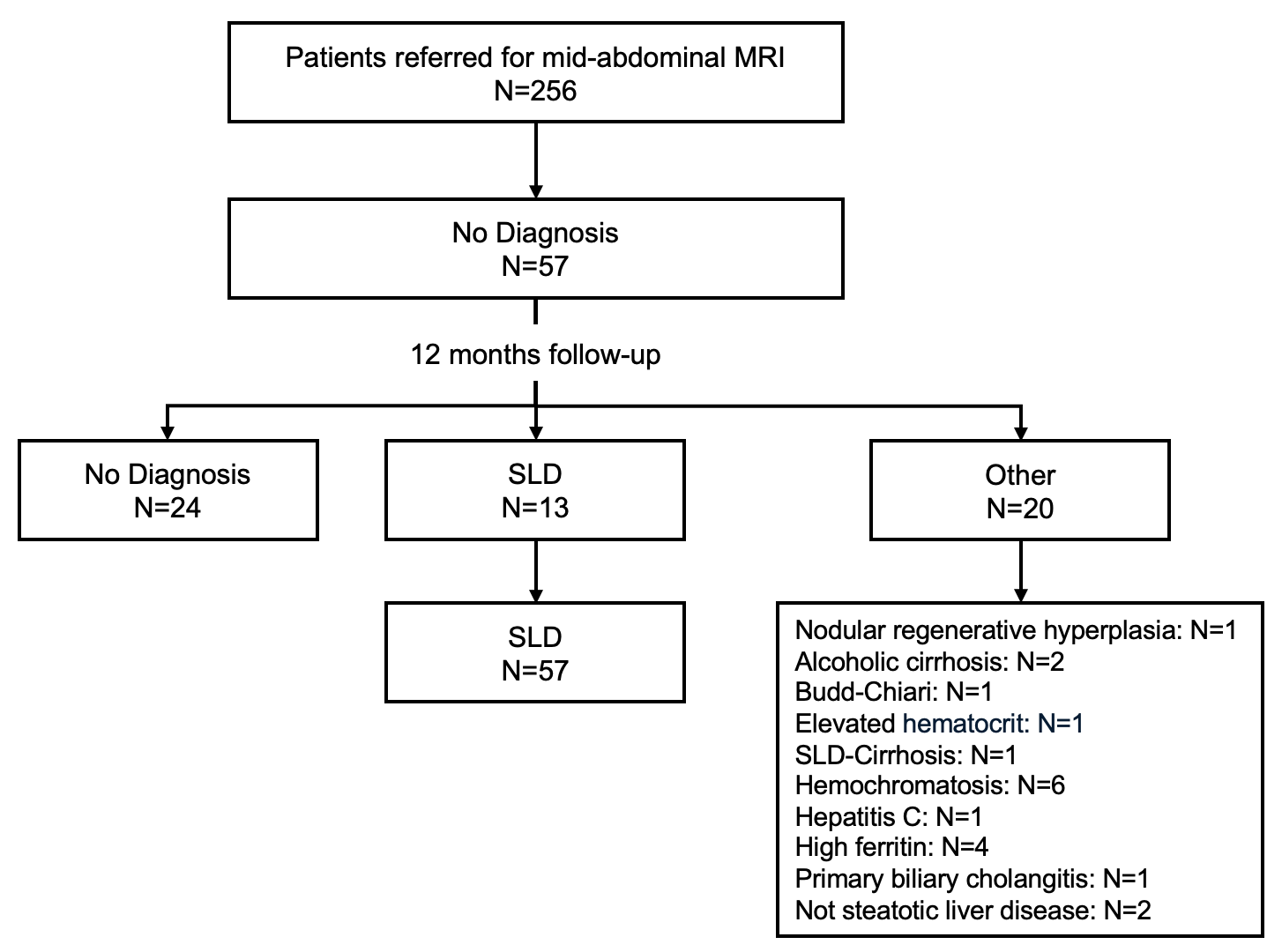

Supplement: Supplementary file 2 — Figure S2: Distribution of patients without diagnosis at baseline and the changes in diagnosis over the follow‐up period. [file LIV-45-0-s002.docx]
