# Supplementary material for: Real‐World Assessment of Liver Corrected T1 and Magnetic Resonance Elastography in Predicting Liver Disease Progression
Source: Liver Int. 2025 Aug 14;45(9):e70280. doi: 10.1111/liv.70280 (PMC12351529; doi:10.1111/liv.70280)

**Supplementary Figure 3**: Longitudinal changes in laboratory biochemical (blood) markers across subgroups defined by cT1 and MRE for those without a diagnosis at both baseline and follow-up.


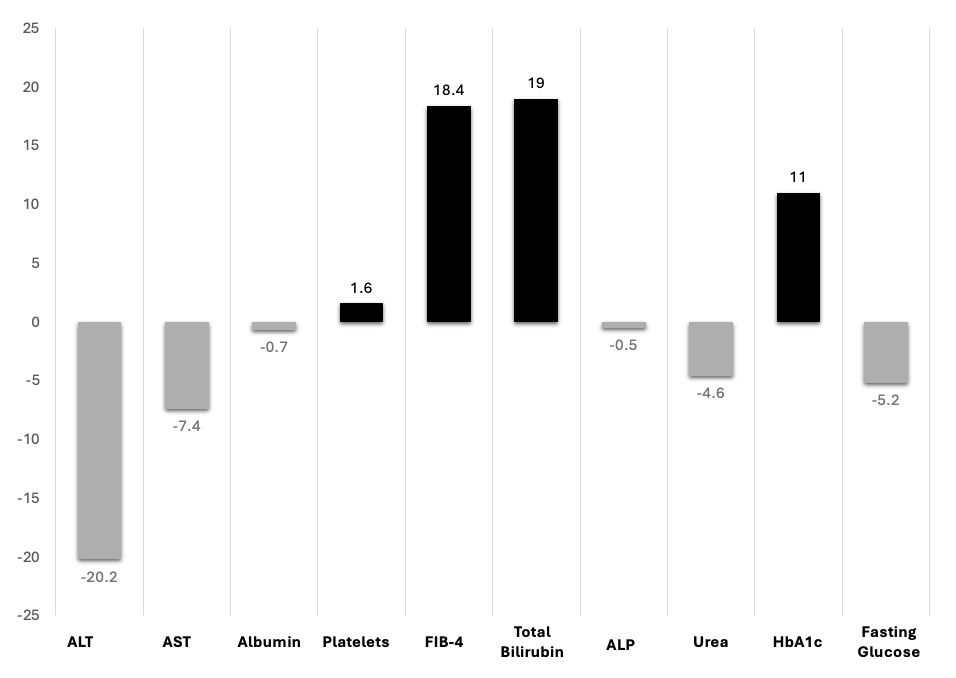

Supplement: Supplementary file 3 — Figure S3: Longitudinal changes in laboratory biochemical (blood) markers across subgroups defined by cT1 and MRE for those without a diagnosis at both baseline and follow‐up. [file LIV-45-0-s003.docx]
