# Supplementary material for: Real‐World Assessment of Liver Corrected T1 and Magnetic Resonance Elastography in Predicting Liver Disease Progression
Source: Liver Int. 2025 Aug 14;45(9):e70280. doi: 10.1111/liv.70280 (PMC12351529; doi:10.1111/liv.70280)
Supplement: Supplementary file 4 — Table S1: Scanner types, scanner parameters and sequence acquisition parameters for LiverMultiScan and MRE. [file LIV-45-0-s001.docx]

**Supplementary table 1:** Scanner types, scanner parameters and sequence acquisition parameters for LiverMultiScan and MRE.

| **LiverMultiScan** | |
| --- | --- |
| Field of view (mm^3^) | 440 x 330 x 100 |
| Reconstruction voxel size (mm) | 1.15 x 1.15 |
| Slice thickness (mm) | 8 |
| Slice gap (mm) | 7 (for liver) |
| Slices | 5 for liver, 1 for pancreas |
| Parallel imaging SENSE factor | 2 |
| Repetition time (ms) | 2.42 |
| Echo time (ms) | 1.05 |
| Flip angle (°) | 35 |
| Acquisition duration | 60 seconds for liver, 12 seconds for pancreas |
| Respiratory compensation | 5 breath-holds for liver, 1 breath-hold for pancreas |
| **MRE** | |
| GE scanner | |
| Sequence | 2D gradient-recalled echo (GRE) sequence with motion-encoding gradients (MEGs) incorporated into the readout direction (GE Healthcare, Waukesha, WI). |
| Vibrations | Acoustic vibrations at 60 Hz were transmitted using an active pneumatic driver system positioned over the right upper abdomen. |
| Elastogram generation | Automatic generation on the scanner console using the vendor-supplied MRE processing software. |
| Siemens scanner | |
| Sequence | 2D spin-echo echo-planar imaging (SE-EPI) sequence with motion-encoding gradients applied along the through-plane (z-axis) direction |
| Vibrations | Mechanical vibrations at 60 Hz were delivered via an external passive driver positioned over the liver. |
| Elastogram generation | Elastograms (wave images and stiffness maps) were generated using the integrated vendor-provided processing pipeline. |
